# Comparative study of population genomic approaches for mapping colony-level traits

**DOI:** 10.1371/journal.pcbi.1007653

**Published:** 2020-03-27

**Authors:** Shani Inbar, Pnina Cohen, Tal Yahav, Eyal Privman

**Affiliations:** Department of Evolutionary and Environmental Biology, Institute of Evolution, University of Haifa, Haifa, Israel; Australian National University, AUSTRALIA

## Abstract

Social insect colonies exhibit colony-level phenotypes such as social immunity and task coordination, which are produced by the individual phenotypes. Mapping the genetic basis of such phenotypes requires associating the colony-level phenotype with the genotypes in the colony. In this paper, we examine alternative approaches to DNA extraction, library construction, and sequencing for genome wide association studies (GWAS) of colony-level traits using a population sample of *Cataglyphis niger* ants. We evaluate the accuracy of allele frequency estimation from sequencing a pool of individuals (pool-seq) from each colony using either whole-genome sequencing or reduced representation genomic sequencing. Based on empirical measurement of the experimental noise in sequenced DNA pools, we show that reduced representation pool-seq is drastically less accurate than whole-genome pool-seq. Surprisingly, normalized pooling of samples did not result in greater accuracy than un-normalized pooling. Subsequently, we evaluate the power of the alternative approaches for detecting quantitative trait loci (QTL) of colony-level traits by using simulations that account for an environmental effect on the phenotype. Our results can inform experimental designs and enable optimizing the power of GWAS depending on budget, availability of samples and research goals. We conclude that for a given budget, sequencing un-normalized pools of individuals from each colony provides optimal QTL detection power.

## Author summary

Genomic mapping techniques are used to map phenotypes to genotypes. Mapping is of general interest in any biological system, including fundamental studies of biological traits, clinical studies of genetic predisposition to disease, and agro- and bio-technological studies of domesticated plants and animals. Typically, such studies associate phenotypic measurements of individuals with their genotypes. Here we evaluate methodological approaches for genomic mapping of phenotypes that are expressed at the level of a group rather than that of individuals. We demonstrate that genomic sequencing of a DNA pool from multiple samples provides increased statistical power within a limited budget. Our results facilitate more efficient use of resources in genomic mapping studies that investigate group-level phenotypes.

## Introduction

Social insect colonies depend on the collective performance of a large number of individual workers. Such collective performance gives rise to extended, colony-level phenotypes such as social immune responses to pathogens and parasites, coordinated defense against intruders, information transfer and processing, a colonial pheromonal odor that facilitates nestmate recognition, alternative social structures, etc. [1–4]. These colony-level phenotypes are the collective output of individual phenotypes and are affected by both the internal state of the colony, as well as other factors in its environment (e.g., climate, neighboring colonies, predators and competitors). The colony-level phenotype may be a simple sum of the individual phenotypes in some cases, or a product of a non-linear function in more complex systems. “Emergent phenotypes” are phenotypes that emerge from the interaction of multiple individuals and are not apparent at the individual level. For example, these include foraging patterns and nest architectures [5,6]. Identifying genes responsible for these higher order traits is a challenging endeavor that warrants multilevel modeling.

In genome-wide association studies (GWAS), polymorphic genetic loci are associated with variation in phenotypic traits. These traits are often quantitative traits, and the genetic elements determining them are known as quantitative trait loci (QTLs). For many decades, researchers used QTL mapping approaches that make use of a mapping population consisting of full siblings, usually from a controlled cross of known parental genotypes. In social insects, this approach was employed mainly in honey bees, which are readily crossed in the lab. For example, lines that were artificially selected for high or low pollen foraging [7] and for high or low hygienic behavior [8,9] were crossed and used for mapping QTLs for these colony-level phenotypes. However, most other social insect species do not readily mate in captivity, which precludes controlled crosses. Conversely, the GWAS approach, which has gained popularity in recent years, allows the use of samples from a natural population. While such samples are typically unrelated, kinship among samples or population structure may lead to inference of false associations. Therefore, GWAS methods that account for kinship and population structure were developed [e.g., 10]. In both GWAS and classic QTL mapping, each individual sample is genotyped and phenotyped, and polymorphic loci are tested for an association with the phenotype of interest. This standard scheme becomes more complicated when one wishes to map colony-level traits. While each colony consists of multiple individual genotypes, the phenotype is a single measurement at the colony level. The GWAS methodology needs to be adapted to address this added level of complexity.

Next generation sequencing methods allow for highly cost-effective genotyping. However, sequencing whole genomes in large numbers is still cost prohibitive despite the continuing and dramatic reductions in sequencing costs over the last decade. A project’s sequencing budget is still a significant limiting factor because sufficient statistical power for QTL detection often requires many thousands of samples [11]. Consequently, several sequencing-based approaches were developed to reduce per-sample costs. One approach is known as Restriction-site Associated DNA sequencing (RAD-seq; also known as genotyping-by-sequencing, GBS). In RAD-seq, restriction enzymes are used to limit the sequencing effort to a subset of randomly distributed loci in the genome [12]. Thereby, sequencing costs may be reduced by 5–10 fold compared to commercial whole-genome sequencing (WG-seq) libraries [13]. Another approach to reduce library construction and sequencing costs is pool-seq: pooling DNA from several individual samples and then constructing and sequencing a single library. Multiple pools are sequenced, and the phenotype of interest is tested for an association with the allele frequency in a given locus in each pool. In some studies, library construction can amount to over 50% of the total costs, which makes pool-seq an attractive approach. Pool-seq allows estimating allele frequencies with an increased accuracy-to-cost ratio relative to sequencing individual samples [14,15].

Allele frequency estimation by pool-seq may suffer from experimental noise and biases in various stages of the protocol, including DNA pooling, library construction, sequencing, and data analysis. Previous studies measured the experimental noise in pool-seq by comparing allele frequencies estimated from individually sequenced samples with allele frequencies in their pooled DNA [14,16,17]. Gautier, et al. [14], Rode, et al. [18] and others developed models of pool-seq that account for pool parameters (number of samples, sequencing depth) as well as the experimental error in pooling individuals with unequal representation. These models can predict the expected accuracy for a wide range of parametrizations. Futschik and Schlötterer [19] used simulation studies to compare the power and accuracy of SNP discovery and allele frequency estimation. They simulated allele frequencies of pool-seq and individual genome sequencing over a wide range of parameters and experimental designs. They evaluated the effect of unequal representation of individuals in the pool on population statistics such as Tajima’s π and Watterson’s θ. However, these simulations did not take into account potential errors and biases in various other stages of library construction and sequencing. Such errors and biases are evident from the abovementioned studies by Van Tassell, et al. [16] and Gautier, et al. [14], who empirically measured allele frequencies in pool-seq. Moreover, none of these studies investigated the implications for the probability of detecting QTL when using pool-seq as opposed to individual sequencing in the context of GWAS.

Several theoretical and empirical studies investigated the application of pool-seq to mapping quantitative traits [20–26]. These studies used pools of samples taken from the tails of the phenotype distribution in a natural population. They demonstrated that greater statistical power may be obtained with a much reduced sequencing budget when using pool-seq. For example, Bastide, et al. [26] used pool-seq to identify two QTLs for a highly heritable pigmentation trait in *Drosophila melanogaster*. They used simulations to show that their approach is mainly suitable for detecting QTLs with high frequency alleles and reasonably large effect. The pool-seq approach in these studies is suitable for individual phenotypes, where each pool consists of samples with similar phenotypic measurements.

The motivation for using pool-seq to map colony-level traits is different: the genotypes of a colony may be assayed as a pool that represents the ‘collective genome’ of the colony, which is then tested for association with the collective phenotype. This scenario is distinct from the abovementioned studies in that there may be substantial within-colony phenotypic variation. More importantly, the pooled individuals are not independent samples from the population. They are related individuals (e.g. sisters or half-sisters), and they are sampled from the same nest/hive so their phenotypes were affected by similar environmental conditions.

Here we describe a comparative methodological study evaluating the contribution of alternative approaches to the statistical power of GWAS for mapping colony-level traits. We used colony samples from monogyne (i.e. having a single queen per colony), polyandrous (i.e. queens are multiply mated) C. *niger* ants for this empirical evaluation. We compared WG-seq vs RAD-seq, individual sequencing vs. pool-seq, and their combinations. Thereby, we provide a broad overview of the expected results of different sequencing approaches. Using simulations, we report the minimum number of individually sequenced samples per colony that is equivalent to a pooled sample in terms of the statistical power of GWAS. Our simulations also take into consideration environmental factors that affect the phenotype to various degrees (i.e. different levels of heritability), which substantially affect the power of the GWAS. We also used the simulation study to generalize our results to different levels of polyandry, as well as monandry (singly-mated queens). Our results enable optimization of experimental designs for GWAS of colony-level traits. We conclude that pool-seq is a significantly more powerful approach for mapping colony-level traits, although depending on availability of samples and scientific goals, individual sequencing may be advantageous in some scenarios.

## Methods

### Model organism

Our study population was sampled from Betzet beach on the northern Israeli coastline. This is a population that was previously described as *Cataglyphis drusus* [27], but our recent species delimitation study suggested that this is the same species as *C*. *niger*, because these populations are not differentiated by their nuclear genomic DNA [28]. Colonies of this population are monogyne (headed by a single queen), polyandrous (queens are multiply mated) and monodomous (single nest per colony) [27]. We chose a monogyne population in order to avoid substructures of the multiple families within a polygyne colony, which would further complicate the analysis. A monogyne, polyandrous population in this species has a mean within-colony genetic relatedness of 0.26 [29].

### Samples and pooling schemes

Thirty workers were sampled from each of three colonies (colony BZT48: N33.05277, E35.10277; colony BZT49: N33.05243, E35.10278; colony BZT50: N33.05162, E35.10245) and every ant was individually frozen in -80°C on the evening of the same day. Workers of all sizes were included in the sampling. DNA was extracted individually from whole bodies, using TRIzol (Invitrogen Life Technologies) and kept in -20°C. Quantity and quality of extracts were determined by NanoDrop 2000 Spectrophotometer and gel electrophoresis was used to verify sample consistency, i.e., DNA yield, degradation, and phenol/salts residues. Pooled samples from individually extracted samples were prepared by normalizing DNA quantities based on NanoDrop measurements, for equal representation of every individual sample in the colony’s pool. Pipetting was done using a manual Gilson pipette (2-20ul).

### RAD library construction

A reduced representation genomic library was constructed according to a modified double-digest Restriction-site Associated DNA sequencing (ddRAD-seq) protocol, based on protocols from Parchman, et al. [30] and Peterson, et al. [13]. Briefly, DNA was digested by two different restriction enzymes (EcoR1 as a rare-cutter; Mse1 as a frequent-cutter) and ligated to barcoded adaptors for multiplexing (using CutSmart buffer NEB). Products were amplified using Q5 Hot Start Polymerase NEB, with the number of cycles reduced to 20, the number of replicates increased to four, and starting DNA volume increased to 6ul. Also, primers and dNTPs were added to the final thermal cycle in order to reduce production of single-stranded or heteroduplex PCR products. Products were separated by gel electrophoresis and insert sizes of 300-400bp were selected and purified (Qiagen, MinElute Gel Extraction Kit, followed by Beckman Coulter, Agencourt AMPure XP). The library was sequenced in one lane of a single-end, 100bp reads, on a HiSeq4000 Illumina sequencer. Sequenced data was submitted to NCBI SRA database (Accession: PRJNA494296).

### Whole genome library construction

In addition, for 29 individuals of colony BZT50, DNA was extracted from three body segments, i.e., heads, thoraces and abdomens, but without legs. DNA was also extracted from all legs pooled together (174 legs). One pooled sample of the 29 individuals was normalized for equal quantitative representation of each sample and two additional normalized pools were independently prepared from only 10 individuals out of the same 29. Normalization was based on NanoDrop measurements. Uniquely barcoded genomic libraries were constructed for each of these 33 individual/pool samples (Illumina TruSeq Nano DNA Library Prep kit). Briefly, with this method, a Covaris S220 sonicator was used for DNA shearing of 550bp inserts, ends were repaired, and the fragments were size-selected and purified using magnetic beads (supplied in kit). Then, an adenosine was added to the 3’ end of the blunt fragments, individually barcoded adaptors were ligated, and ligated fragments were amplified (eight PCR cycles). Libraries were normalized and pooled so that the four pools were represented five-fold more than individual samples. The library was sequenced in one lane of a paired-end, 150bp reads, on a HiSeq4000. Sequenced data was submitted to NCBI SRA database (Accession: PRJNA494296).

### RAD-seq data analysis

Samples were analyzed by STACKS [31,32] and BWA [33] to create a catalogue of single nucleotide polymorphism (SNPs). Sequencing was in high quality (Mean Phred scores ranged between 30–40 for all bases i.e., a chance of 1 to 1000 for sequencing error at worst case scenarios). The number of raw reads totaled in 302,654,900, of which 178,631,035 were associated with a sample (barcoded). 166,681,127 reads remained after filtering low-quality reads using process_radtags from the STACKS package. The average depth per sample of the retained reads ranges between 0 and 132X, averaging at X13.7 per sample (calculated based on 67,687 restriction sites in the reference genome). Reads were aligned to the *C*. *niger* reference genome version Cnig_gnA (NCBI accession number pending) using BWA-MEM and those with low alignments scores or multiple mapping were filtered out, leaving 121,343,143 reads (See Table 1). Remaining reads were analyzed together to create a catalogue of all alleles found using the STACKS pipeline, resulting in a catalogue of 50,139 SNPs.

**Table 1:** Sequencing reads per colony before and after initial processing and after alignment based filtering.

Colony	Total reads	Retained reads after initial processing	Average retained reads per sample
48	76,686,117	72,331,894	1,859,007
49	54,838,668	50,950,729	1,294,269
50	47,106,250	43,398,504	1,170,825

### Whole genome data analysis

Sequences were analyzed using BWA and GATK [34] to create a catalogue of SNPs. Sequencing was in high quality (Mean phred scores ranged between 20–30 for all bases). The number of raw reads totaled in 675,094,588 (2 X 337,547,294), of which 628,874,982 (2 X 314,437,491) were associated with a sample. Duplicated reads were removed (i.e., multiple read pairs that were identical in their sequence) and low quality reads were filtered leaving 614,306,762 reads (97%). Low quality reads were trimmed or removed using Trimmomatic software, leaving sequences that were at least 75bp long. The total number of remaining reads was 529,101,399 (average depth 6.4±2.2X per sample based on genome assembly size of 296,608,662bp). Reads were aligned to the reference genome using BWA and 502,721,013 reads remained after filtering low-scoring alignments and reads with multiple mapping (74% of raw reads).

Genotypes of individual samples were inferred using the standard GATK pipeline [34]. Briefly, SNP calling was performed using HaplotypeCaller and GenotypeGVCFs from the GATK package, using the Bayesian approach. For pooled samples, the proportions of reads supporting each allele were calculated at each locus (based on the AD field in the VCF output from GATK). Allele frequencies of the pool sample were compared to the frequencies in the individual genotypes. GATK produced a catalogue of 2,000,424 SNPs and 1,459,065 SNPs remained after removal of indels and SNPs with more than 2 alleles. SNPs with excessive depth were removed because they are suspected as repetitive sequences, using a threshold of 450X total depth across all samples (which was two standard deviations above the mean depth) leaving 1,386,753‬ SNPs.

### SNP filtering

We tested a range of parameters configurations for SNP filtering. A minimal number of reads covering a SNP (*m*) is required to call the genotype of a particular sample in a particular SNP locus. We set a higher minimum for the pooled sample (10, 15, 25, or 30) than for individual sample (2, 4, 6, or 8). SNPs were required to have called genotypes for a minimal percentage of individual samples (*r*), ranging between 75 and 95, and a minor allele frequency (MAF) of at least 1.25%, 3%, or 9% (*min_maf*). For the WG-seq dataset that contained fewer samples (from only one colony) we implemented an alternative to the MAF cutoff: we required that the minor allele appear in at least two samples. Thus, we permit cases of two heterozygous, but not a single homozygote for the minor allele. A total of 400 different filtering options were tested. Spearman ranked correlations were calculated between the individuals and each of the pooled samples. The following filtering criteria were chosen based on the higher correlation obtained between the pooled and the individual genotypes, so long as the filtering did not discard most of the SNPs (see Results). For the RAD-seq data, filtering criteria were: *r* = 90%, *min_maf* = 3%, *m* = 4 for an individual sample and *m* = 10 for a pooled sample. Filtering criteria for WG-seq were: *r* = 75%, *min_maf* = 1.25%, *m* = 2 for an individual sample and *m* = 10 for a pooled sample.

### Simulations

**Fig 1:**
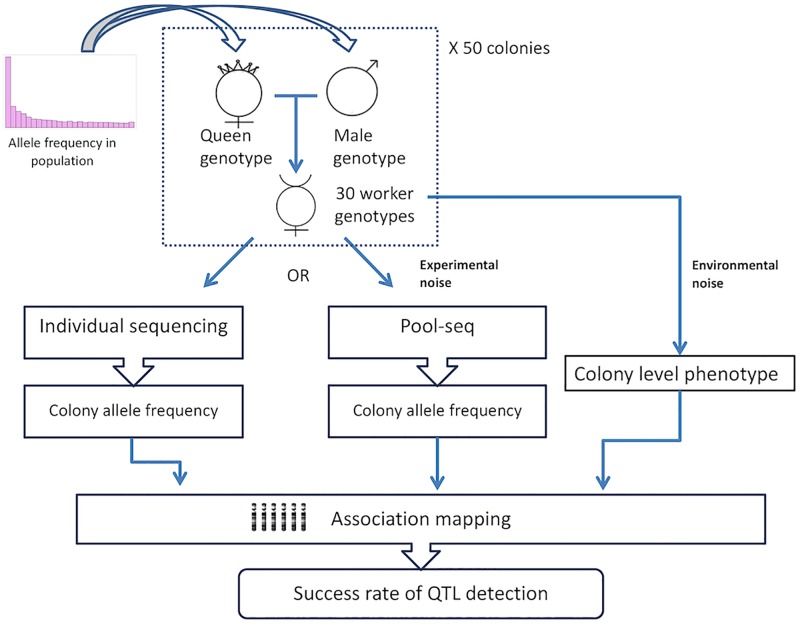
Simulation scheme. Allele frequencies of 10,000 SNPs are sampled from an empirical distribution. Genotypes were randomized for the mother and five fathers of each colony, and then for 30 of their daughter workers. The corresponding pool allele frequencies were sampled according to the empirical correlation between pools and individuals. Colony level phenotypes were generated based on the individual genotypes at a single phenotype-affecting SNP and at varying environmental noise levels. The success rate for detecting the causal SNP was determined for 100 simulated datasets.

The purpose of the simulations was to compare the power of GWAS using the pool-seq approach to that of sequencing of individual samples. Fig 1 shows a flowchart of the simulation scheme. For a realistic allele frequency distribution, we randomly sampled 10,000 SNPs from an empirical SNP dataset of 310 RAD sequenced *Solenopsis* fire ants [35]. Based on the allele frequencies of the sampled SNPs, we randomly sampled genotypes for the queens and her single mate (monandrous) five mates (as in *C*. *niger*) or ten mates, for 50 simulated monogyne colonies (that is, if a given fire ant SNP had allele frequencies of *p* and 1-*p*, then each of the simulated alleles for the queen and her mates was randomly drawn using these probabilities). Note that there is no population structure in our simulations since each colony is an independent sample of genotypes. Then, genotypes of 30 workers were randomly generated for each colony based on the parents’ genotypes. Three replicates of pool-seq results (for the 30 worker samples of each colony) were simulated according to the empirically observed relationship between the individual genotypes and the pool allele frequency in our WG-seq data from *C*. *niger* (above). Specifically, for every SNP in a simulated colony we randomly chose a SNP in the *C*. *niger* data that had a colony allele frequency (estimated based on individual sequencing) that was similar to the allele frequency in the simulated colony, and we assigned the respective *C*. *niger* pool-seq allele frequency as the simulated pool-seq allele frequency. Finally, we designated a single SNP as a QTL—a causal SNP affecting a single quantitative trait at the colony level. The trait of interest was affected by the genotypes of the colonies such that one allele gives a higher phenotypic value and the other gives a low value. If all the colony was homozygous to the “high” allele than its colony-level phonotype was 1 unit higher than the colony-level phenotype of a colony entirely homozygous to the “low” allele. Intermediate values were assigned based on the proportion of “high” alleles in the colonies (i.e. assuming an additive model). We conducted association tests to evaluate power for detecting that QTL. We examined 30 alternative scenarios for mapping the QTL using the genotypes of between 1 and 30 individuals per colony. Additionally, we examined the power of using the average of triplicates of pools from each colony.

Simulation were done under the assumption that there is no linkage disequilibrium between the simulated SNPs. Environmental factors affecting the quantitative trait were accounted for by adding varying levels of “noise” to the phenotype—a randomly chosen number from a normal distribution with a mean of 0 and standard deviation of 0, 0.5, 1, 1.5, 2, 2.5 or 3. To measure the resulting ratio between the genetic and environmental factors we empirically measured the percent of phenotypic variation explained by the genotype in a linear regression analysis (the R^2^ of the regression).

For each simulation scenario with a given level of noise, we simulated 100 data sets of 50 colonies and 100 sets of 150 colonies. Each time, a single SNP was chosen out of the 10,000 SNPs, and 10 phenotypes were simulated using a given level of environmental noise. In total, 2000 simulated data sets were generated for each noise level. We attempted to predict which of the 10,000 SNPs affects the phenotype using linear regression (implemented in the R function *lm*) in each of the scenarios: 1,2,…, or 30 individuals per colony, or a pool for each colony (with either 50 or 150 colonies). When dealing with real population samples, GWAS methods need to use a linear model that accounts for population structure and/or kinship [e.g., 10]. However, in our simulations each colony is an independent sample from the general allele frequency of the population, so a simple linear regression is adequate. A prediction was determined as successful when the QTL was identified with *p*-value < 0.05, after false discovery rate (FDR) correction for 10,000 tests [36].

## Results

### Allele frequencies and their correlations between pool-seq and sequencing of individual samples

Individual samples from three colonies (30 per colony) were extracted separately and yielded DNA concentrations of 44±32 ng/ul (mean and standard variation; S1 Table). Normalized pools were constructed based on the measured DNA concentration for each sample. A non-normalized pool was also obtained by extracting DNA from the legs of 29 ants together in one tube. Note that the estimated variation in DNA concentration does not fully represent the experimental error because of DNA quantification errors and pipetting errors during the construction of normalized pools, as well as additional errors and biases in subsequent stages of library construction, sequencing, and data analysis. Thus, sequencing of normalized pools will still produce uneven individual representation with unknown variation. Individual samples and three types of pools were analyzed by WG-seq: a normalized pool of 29 individual samples, two normalized pools of 10 samples (out of the same 29), and a non-normalized pool of legs (from the same 29 samples).

SNPs were identified and genotypes were called for each individual sample. For the pools, allele frequencies were calculated based on the read count for each allele. These results were used to calculate the correlation between allele frequency estimates from the pool vs. the individual sequencing. Depending on SNP and genotype filtering criteria, *rho* values ranged between 0.9 and 0.97 (Spearman), but the highest correlation was achieved with very strict filtering criteria that left very few SNPs. Therefore, we chose a more relaxed filtering criteria: minimum of 4 reads covering a SNP in individual samples and 10 reads in the pool sample, and no more than 25% of missing data per SNP. The different pooled samples showed similar *rho* values, between 0.931–0.938, and the number of SNPs were between 833,080–833,479 (Table 2). Surprisingly, the normalized pools did not produce a better correlation than the unnormalized pool (of legs).

**Table 2:** Correlations between allele frequencies in individual genotypes and pool-seq.

Colony/pool type	Individual depth (raw reads; average and std. dev.)	Pool depth (raw reads)	Number of SNPs	Correlation
RAD-seq Colony 48	26 ± 38X	3.7X	2,232	0.709
RAD-seq Colony 49	12 ± 10X	6.0X	2,966	0.774
RAD-seq Colony 50	11 ± 11X	19X	3,254	0.770
WG-seq normalized pool-10-1	6.4 ± 2.2X	34X	833,463	0.938
WG-seq normalized pool-10-2		38X	833,479	0.931
WG-seq normalized pool-30		27X	833,080	0.937
WG-seq unnormalized pool-legs		26X	833,208	0.938

**Fig 2:**
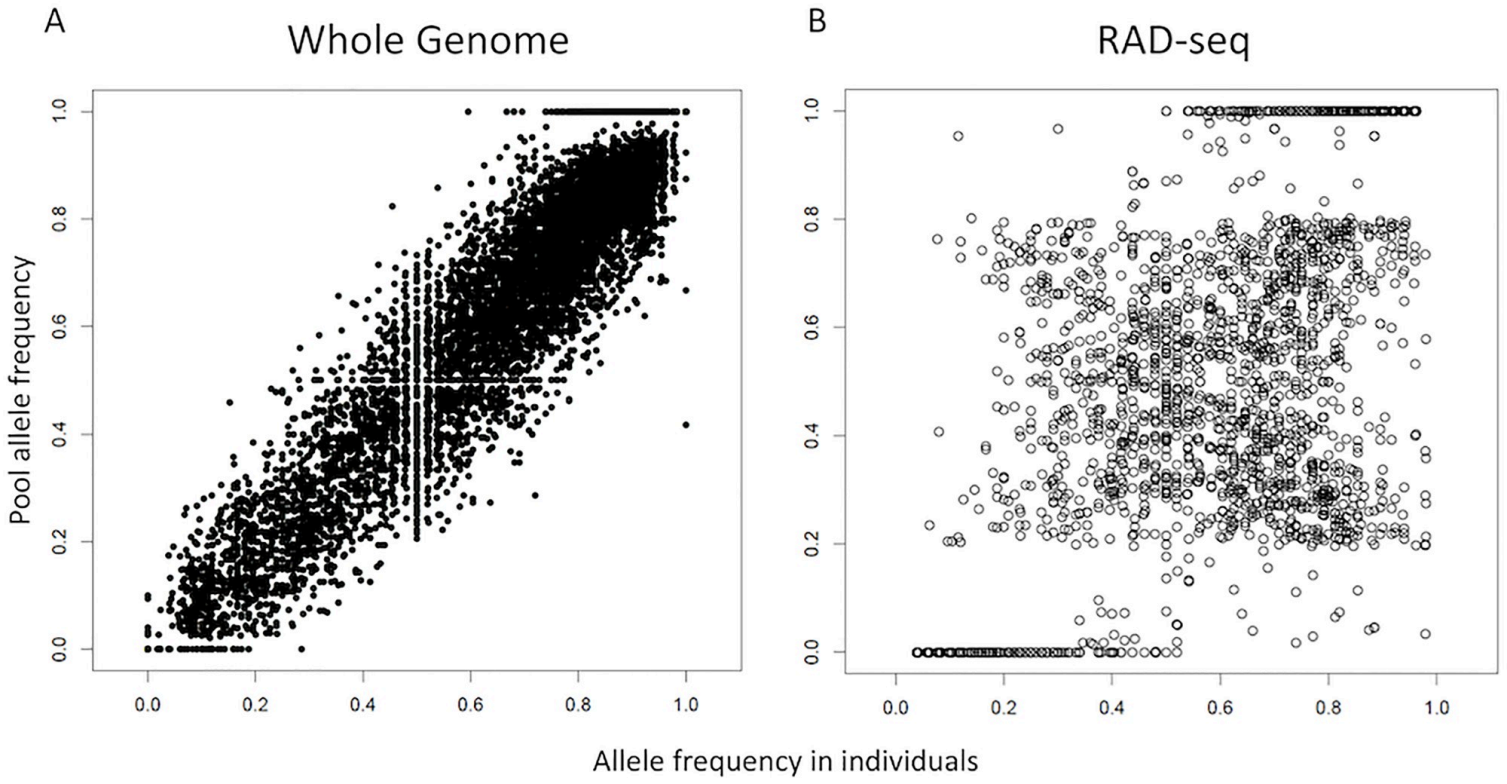
Allele frequency plots of whole genome sequencing (A) and RAD-seq (B) of pooled samples vs. individual samples from colony 50.

Similarly, we chose these filtering criteria for the RAD-seq data: a minimum number of 4 reads for individual samples and 10 reads for pooled samples, 10% missing data per SNP, and 3% minimum minor allele frequency. The estimated correlation between pools and individual sequencing was much lower than that of WG-seq, with *rho* values between 0.709–0.74. The much lower correlation in the RAD-seq data compared to the WG-seq data can also be clearly seen in plots of the allele frequencies estimated by pool-seq vs. those estimated from the individual genotypes (Fig 2).

We investigated the effect of sequencing depth on the accuracy of pool-seq allele frequency estimation by sub-sampling the WG-seq reads by a factor of 2/3, 1/2, or 1/3. We repeated our analysis of the sub-sampled raw sequencing data and the evaluation of correlation between the pool and the individuals. The reduced depth resulted in smaller number of SNPs passing our filtering criteria, as well as lower correlation. Compared with the correlation coefficient of 0.938 over 833208 SNPs at the original depth of 26X (for the unnormalized pool), sub-sampling by a factor of 2/3, 1/2, or 1/3 resulted in correlation coefficients of 0.913, 0.838, or 0.786 over 179073, 22634, or 12086 SNPs, respectively (S2 Table).

For both RAD and WG libraries, a comparison of the allele frequency distributions between individual sequencing and pool-seq data shows that the pool-seq analysis assigns 100% allele frequency to many alleles that have less than 100% frequency in the individual genotypes (Fig 2). Furthermore, there is a gap with very few SNPs between 80% and 100% allele frequency in the RAD pool-seq allele distribution, and between 95% and 100% in the WG pool-seq. Conversely, the individual genotypes show that many SNPs should have allele frequencies in this range.

**Fig 3:**
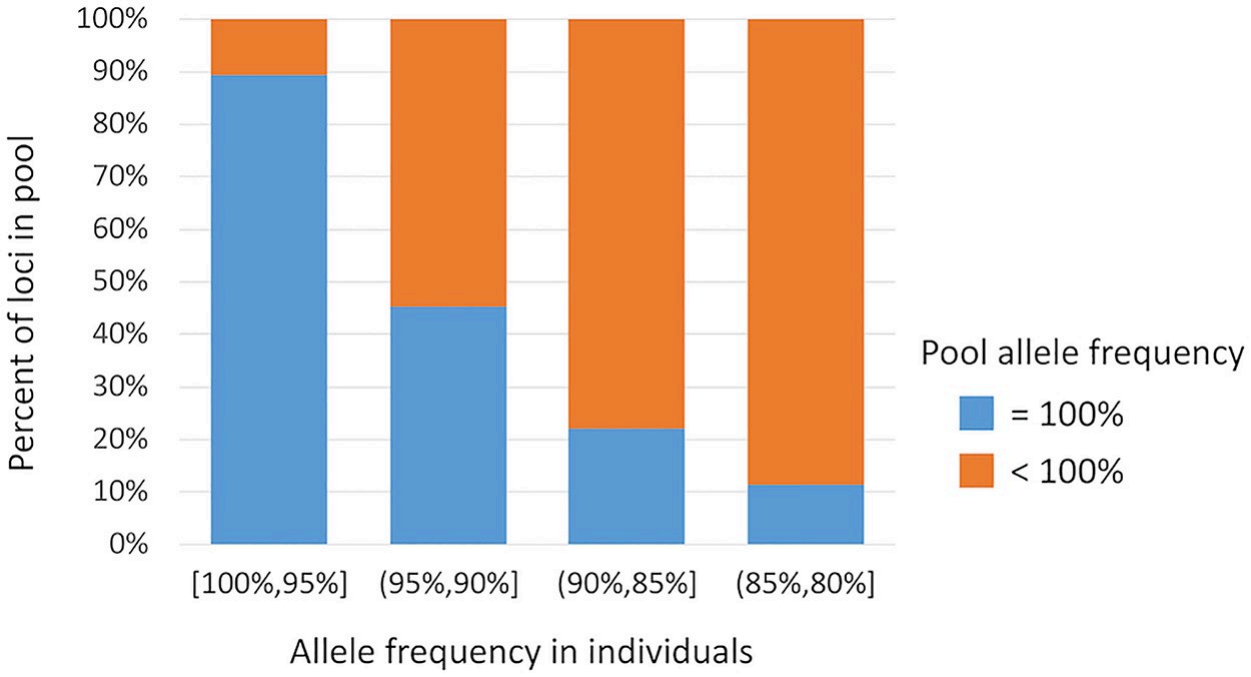
Allele dropout in pool-seq. Loci were binned according to allele frequency in individual samples, and then categorized by whether the allele frequency from pool-seq was 100% (blue) or not (orange). That is, blue indicates allele dropout where the minor allele was not detected by pool-seq.

To investigate this gap, we plotted in Fig 3 the proportion of SNPs with 100% pool-seq allele frequency for bins of individual allele frequency (for pool-10-1). Looking at the subset of SNPs with individual allele frequency above 95% and below 100%, the large majority of these have a pool allele frequency of 100%: 70,826 out of 79,190 (89.4%). We suspected that the loci with 100% pool allele frequency had lower sequencing depth than those with less than 100%, but this turned out not to be so. The 100% pool frequency loci had an average depth of 30.46X, 36.13X, 40.00X, and 28.37X in the four WG-seq pools, while the loci with less than 100% pool frequency had slightly lower average depth of 29.39X, 35.03X, 38.78X, and 27.39X, respectively. Therefore, the difference in depth cannot explain the observed allele dropout in pool-seq.

### QTL detection power for GWAS using pool-seq

**Fig 4:**
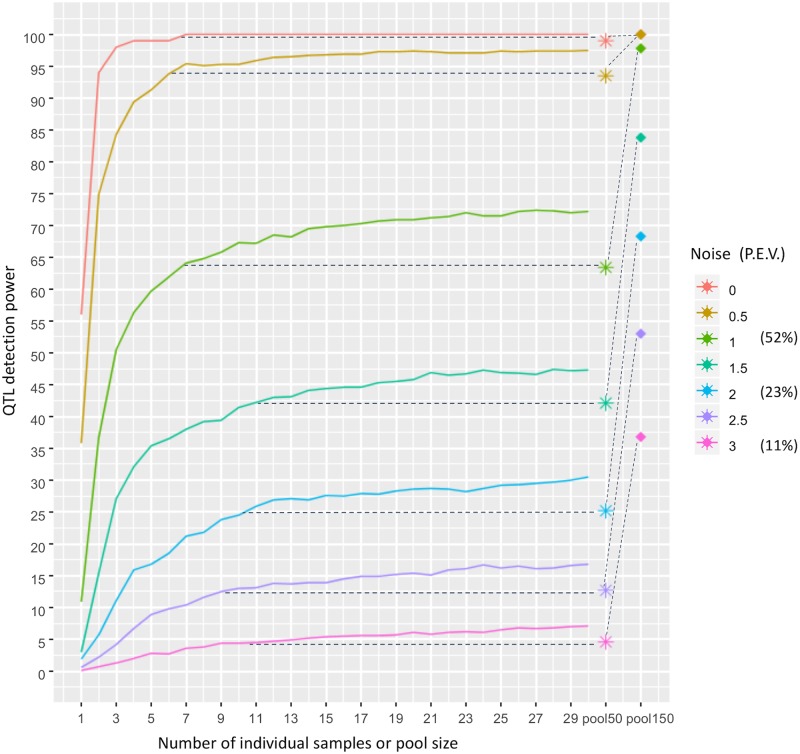
Power of QTL identification. Success rate for detecting a QTL with p<0.05 in 1,000 simulations of 50 colonies (or 150 colonies in the case of the “pool-150” results), with individual sequencing of 1 to 30 samples or pool-seq, and with varying levels of environmental noise. Different colors show different levels of noise that affect the phenotype (P.E.V. = percent explained variation; i.e. the percent of phenotypic variation that is explained by the QTL). The dotted lines illustrate the number of individual samples that provides equivalent power as a pool when only 50 colonies are used, whereas pools for 150 colonies provide much greater power as shown by the diagonal dotted lines.

Next, the statistical power of QTL detection was evaluated in a simulation study, for each of the various approaches described above. Genotypes of workers from 50 colonies were simulated, as well as simulated pool-seq allele frequencies that incorporated the experimental error as measured from the *C*. *niger* whole-genome sequencing data. One SNP was assumed to be a QTL of a single colony-level phenotype. Various levels of noise were added to the phenotype, to represent an environmental effect on top of the genetic effect of the QTL. For example, a noise level of 1 means that the environmental effect is on the same order of magnitude as the genetic effect of the QTL. The percent of phenotypic variation explained by the QTL was measured by linear regression. For noise levels of 1, 2, and 3, the median percent of phenotypic variation explained by the genotype was 52.4%, 23.1%, or 11.5%, respectively (S1 Fig). A linear regression test was used to detect the association of the phenotype with the genotypes of a set of individual samples or with the allele frequency estimated by pool-seq. When using 30 individual samples, the detection rate decreases from 100% with no environmental noise to 7.1% with noise level of 3 (Fig 4). Also as expected, success rate increases with the number of individuals used, but it plateaus for five or more individuals at low noise levels and for 12 or more individuals at the highest noise level. The false positive rate was very low, with only 469 false positive inferences out of 1,000,000 simulated SNPs (SNPs with *p* value < 0.05 after FDR correction; noise level of 1).

The power of pool-seq (63.4% with noise level of 1) is on par with the power of sequencing seven individuals (64.1%). In this case, sequencing seven or more individuals per colony would provide greater power than pool-seq. As noise increases, the pool’s power is equivalent to the power of a larger number of individuals. For example, for a noise level of 2 it is equivalent to eleven individuals. However, if pools of 150 colonies are used, the power of pool-seq increases dramatically to 97.8% for a noise level of 1 (150 pools would be possible within the same budget of sequencing six individuals per colony for 50 colonies; see cost analysis below).

**Fig 5:**
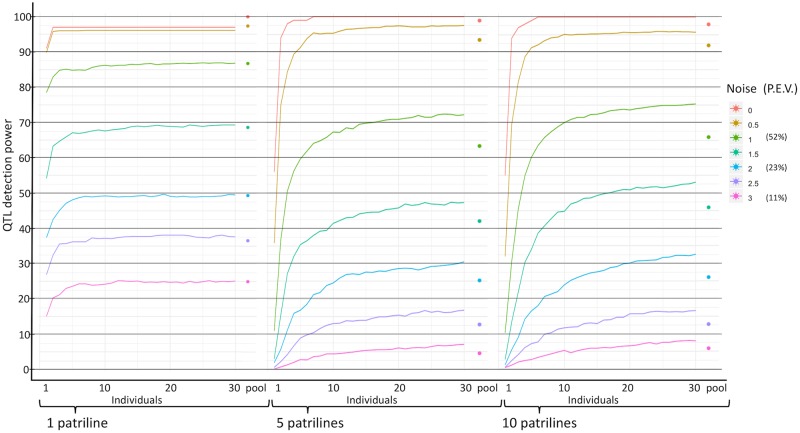
Power of QTL identification for different levels of polyandry (or monoandry). Similar to the simulations in Fig 4, except the queen of each colony has either one, five, or ten mates, i.e. one, five, or ten patrilines per colony (the central plot is identical to the one in Fig 4).

The simulation results presented in Fig 4 are based on the case of *C*. *niger*: monogyne, polyandrous colonies with five matings per queen. To investigate the effect of polyandry, we repeated the simulations for the case of monandry (i.e., a single father for all the workers in the colony), as well as for the case of higher polyandry with ten matings per queen. Fig 5 shows the results for the three levels of polyandry plotted side by side. There are very slight differences between the two levels of polyandry. The general patterns are also similar in the monandrous simulations, but the success rates are generally higher in the monandrous scenario and they reach a plateaus quicker, for a smaller number of individual samples (Fig 5). For example, at a noise level of 1, power plateaus at 87% in the monandrous simulations compared to 72% in the polyandrous simulations. The monandrous simulations reach maximum power already with 9 individuals, whereas the polyandrous simulations do so only with 23 individuals.

### Cost effectiveness

**Fig 6:**
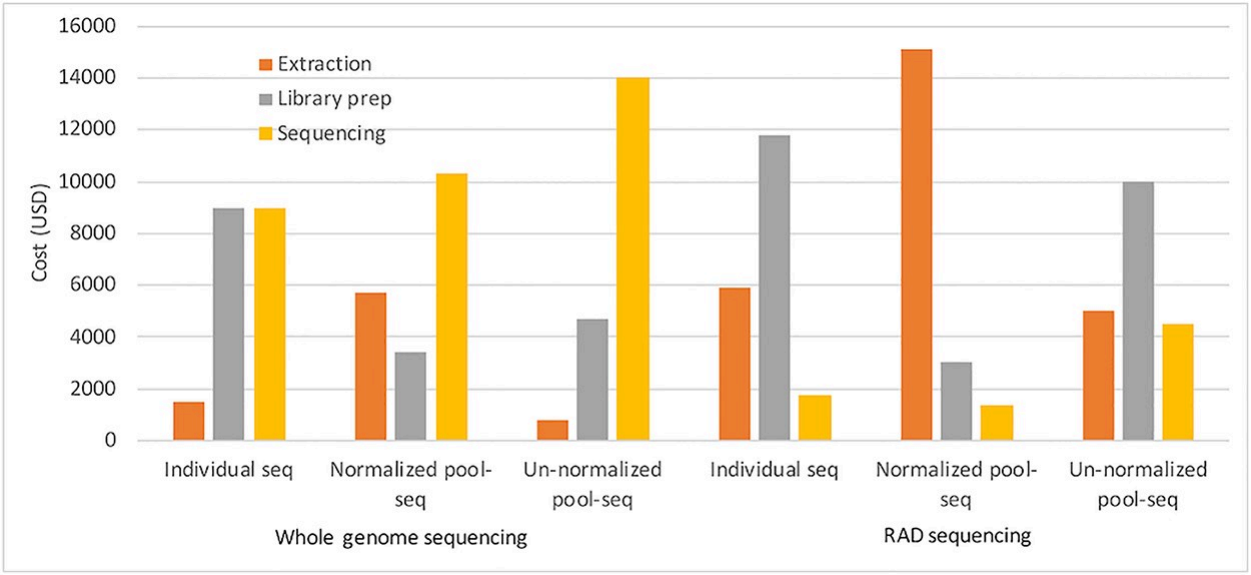
A distribution of costs for a given budget when using different DNA extraction, library construction and sequencing methods.

To aid future studies in determining what would be the favorable DNA extraction, library construction and sequencing approach, we estimated the costs for the various alternatives (Table 3). WG-seq of six individuals from each of 50 colonies would require a budget of approximately 19500 USD (naturally, the actual costs vary from one country to another). We then calculated the number of colonies that could be sequenced using the alternative approaches for the same budget. For this budget, the number of colonies that can be analyzed by pool-seq ranges up to 156 or 1000 colonies for WG-seq or RAD-seq, respectively (when individuals are pooled before DNA extraction). These estimates assumed a desired depth of 10X per individual sample and 30X for pool sample. The costs include the number of DNA samples extracted and the number of libraries constructed in each approach. The proportion of budget used for each expense component is shown in Fig 6. One may also consider the difference between the alternatives in terms of labor. Extracting DNA and constructing libraries in large numbers is rather labor intensive, and this is not represented here as a cost. This analysis can help future studies of colony-level traits to choose a combination of sampling, pooling and sequencing approaches which best fit the scientific goals.

**Table 3:** The number of colonies which can be included is a GWAS for a given budget, in the alternative approaches.

		Colonies	Samples	Extraction cost	Libraries	Library prep cost	Sequencing cost	Total cost
**WG- seq**	Individual seq	50	300	1,500	300	9,000	9,000	19,500
	Normalized pool-seq	115	1,147	5,735	115	3,441	10,324	19,500
	Un-normalized pool-seq	156	156	780	156	4,680	14,040	19,500
**RAD-seq**	Individual seq	197	1,182	5,909	1,182	11,818	1,773	19,500
	Normalized pool-seq	302	3,023	15,116	302	3,023	1,360	19,500
	Un-normalized pool-seq	1,000	1,000	5,000	1,000	10,000	4,500	19,500

Cost estimates in USD. Assuming 5 USD per DNA extraction; 30 USD per WG library construction and 10 per RAD library; 10 USD per Gb sequencing; 3 Gb for a genome depth of 10X per individual sample or 9 Gb for 30X per pool; and 20-fold less sequencing for RAD-seq relative to WG-seq.

## Discussion

In this study, we report the first use of empirical measurements of experimental error in pooling DNA from multiple individuals for the purpose of evaluating the power of GWAS for detecting QTLs of colony-level traits. By evaluating the use of pool-seq vs. individual sequencing and WG-seq vs. RAD-seq, our study provides a broad overview on the experimental errors and biases in allele frequency estimations. Such pool-seq experimental biases can result from DNA quantification errors before normalization, pipetting errors, and various biases and artifacts in library construction and sequencing. We quantified the correspondence between allele frequencies in the individual genotypes and the pool-seq data by their correlation. Using simulations, we estimated, for the first time, the statistical power for detecting a QTL of a colony-level trait by taking into consideration the additive effects of environmental noise and experimental noise.

WG-seq of pools provides considerably more accurate allele frequency estimates than RAD-seq of pools. The correlation between allele frequencies in individuals and in pool-seq was *rho* = 0.94 for WG-seq and *rho* = 0.77 for RAD-seq. Surprisingly, the correlation was not affected by either pool size (30 vs. 10 pooled individuals) or normalization following DNA extraction. It may be that DNA quantification error and pipetting errors did not allow for sufficient reduction in the variation of sample representation in the pool. Furthermore, subsequent stages of the library construction, sequencing and bioinformatic analysis may contribute to the error more than the initial variation in DNA extraction yield. For example, library construction includes PCR amplification that introduces variation in the amplification of different samples, and may also introduce bias toward one allele vs. the other.

We evaluated the importance of sequencing depth by sub-sampling our sequencing depth of the pool from approximately 30X down to 20X, 15X, and 10X. The reduced accuracy of the pool-seq allele frequency that we observed, from 0.938 to 0.913, 0.838, and 0.786, suggests that 30X is advantageous over 20X, but not by a large margin. It appears that pool sequencing depth of 30X is close to the asymptotic limit and further increase in depth (e.g. 40X) would produce only a minor improvement in the accuracy of allele frequency estimation by pool-seq. This conclusion is broadly in agreement with previous pool-seq studies—see comparisons below.

The weaker correlation in the pools of RAD-seq relative to WG-seq may be due to RAD-seq artifacts. For example, polymorphism at restriction sites would result in alleles that are not cleaved and sequenced (termed “allele dropout”), which would bias allele frequency estimation for adjacent SNPs. This effect was shown to result in up to 9.4% of heterozygous loci being incorrectly called as homozygous in RAD-seq of individual samples [37,38]. Gautier, et al. [38] suggested that filtering loci with reduced sequencing depth might partially mitigate this issue. To see whether RAD-seq data at any particular locus is indeed inferior to WG-seq data, we reanalyzed the intersection of loci that were genotyped by both methods (1586 SNPs in colony 50). Also for this reduced dataset, there was an advantage to WG-seq (*rho* = 0.932) over RAD-seq (*rho* = 0.699), which was very similar to the difference that was measured for the full dataset. Therefore, the difference is due to the lower quality of the pool genotype data at each locus that is generated by RAD-seq vs. WG-seq.

Our study also revealed what appears to be a technical artifact in pool-seq. For low frequency alleles (<5% of the colony genotypes), pool-seq misses the rare allele completely. That is, no reads were sequenced from the rare allele in the pooled samples and so the allele frequency estimate is 100%. This cannot be explained simply by low sequencing depth, since the pool had an average sequencing depth of 30X. At this depth, the probability of not obtaining any reads for an allele with 3% frequency is only 0.97^29^ = 0.41. Thus, we conclude that technical artifacts and biases in the pool-seq procedure lead to missed rare alleles in almost all such loci. Our analysis of these loci show that they do not suffer from lower sequencing depth compared to loci that were not affected by allele dropout. Therefore, we speculate that some other technical artifacts in library construction and sequencing introduce noise and/or bias towards one of the two alleles at each locus, which result in rare alleles being completely missed below a certain allele frequency (for a given level of sequencing depth). It should also be noted that pooling methods are disadvantageous when genotyping dominant/recessive markers. In such cases, pool-seq masks heterozygosity whereas individual genotyping enables distinguishing between two heterozygotes and a recessive homozygote.

Evaluation of QTL detection power in GWAS revealed that as the environmental noise level rises, the success rate of identifying a colony-level QTL drops dramatically. Note that our simulated noise levels 1–3 correspond to QTLs that explain between 10% and 50% of the phenotypic variation. This range includes, for example, two QTLs for pollen foraging that were mapped in honey bees, which had effect sizes of 38% and 33% [7]. We conducted this simulation study for samples from 50 colonies, which is on the low end of the number of samples typically used for GWAS and QTL mapping studies. Naturally, increasing the number of colonies results in increases QTL detection power, and allows detection of QTLs of smaller effect size. Our simulated consisted of only 10,000 SNPs, and not millions of SNPs that are often genotyped in a typical human GWAS. This is because mapping studies in non-model insect species cannot usually discover so many SNPs due to the limited sample size and smaller genomes. Studies that include more SNPs would be affected by the multiple testing correction to a greater extent, which would reduce the QTL detection power. We also note that QTL detection in our simulation study did not rely on linkage between the genotyped loci and the QTL. When using approaches that genotype only a subset of the loci in the genome (such as RAD-seq or SNP microarrays) it is important to consider the expected linkage between the genotyped loci and the actual QTL. However when using whole-genome sequencing one can assume that the QTL itself is genotyped directly, so we did not include linkage in our simulations.

Our results show that sequencing more individuals per colony can compensate for poor QTL detection power, but only to a limited extent. For example, at a noise level of 1, sequencing seven individuals per colony gives a detection power of 64.1%, but larger numbers of individuals per colony increase the power by less than 10% (Fig 4). When using 50 colonies, the power of pool-seq is equivalent to the power of a sequencing a small number of individual samples—between six and eleven individuals per colony, depending on the environmental noise. However, the reduced costs of pool-seq allows for increasing the number of colonies in the study from 50 to 156 within the same budget when doing WG-seq, and to 1000 colonies when doing RAD-seq. Thus, pool-seq has two opposite effects: reducing the accuracy of allele frequency estimation for each colony, while increasing the number of colonies. The combined result is a dramatic increase in detection power when using pool-seq. For example, at a noise level of 1, sequencing seven individuals per colony, for 50 colonies, gives a detection power of 64.1%, while pool-seq of 150 colonies increases the power to 97.8%. A caveat of this analysis is that we assumed an additive model whereby the effect on the phenotype is a linear function of the allele frequency in the colony. Other genetic models including dominance and other non-linear relationships may lead to different results.

We have concentrated on the case of a monogyne, polyandrous colony with five patrilines (five mating per queen). However, the level of polyandry is variable in social insects, with many monandrous species (singly mated queens) and some extreme cases of polyandry with 20 patrilines or more (e.g. honey bees [39,40]). The level of polyandry in a monogyne colony is indeed the main determinant of genetic diversity within the colony. In monandrous colonies (of monogyne, haplodiploid species) there is only a very limited set of possible allele frequencies (in a bi-allelic locus): 0%, 25%, 50%, 75%, 100%. Conversely, polyandrous colonies with five or more patrilines have many more possible values of allele frequencies (e.g. 10%, 35%). Our simulations for the cases of one, five, or ten patrilines (Fig 5) show that increased polyandry beyond five patrilines does not make a substantial difference. Conversely, the QTL detection power in monandrous colonies is substantially higher than in polyandrous colonies, and a smaller number of individuals is needed to achieve optimal power. For example, at a noise level of 1, while seven individuals per colony may be a good choice for polyandrous colonies (with five patrilines or more), only three individuals per colony may suffice for monandrous colonies (i.e. they would already produce nearly optimal power). The power of pool-seq is also different in the monandrous case. With monandrous colonies, the power of pool-seq is close to the optimal power that can be achieved for any number of individuals per colony, whereas pool-seq of polyandrous colonies had lower power than 20 or 30 individuals per colony.

Overall, our results demonstrate that using colony pools facilitate substantial power increase within a given budget. In general, it would be our recommendation to use pool-seq. Pool-seq is more profitable for the case of polyandrous colonies, but also profitable to a lesser degree for the case of monandrous colonies (because a pool is equivalent in power to a smaller number of individual samples). However, in some scenarios, individual sequencing may be still be preferable. For example, if the number of sampled colonies is limited then individual sequencing would provide greater power in the polyandrous scenario (e.g. sequencing seven or more individuals gives greater power than pool-seq if only 50 colonies are available, at noise level 1 in Fig 4). Individual genome sequencing may also be desirable when there are additional data studied at the individual level, such as RNA sequencing for the purpose of expression QTL (eQTL) analysis [41].

Apart from comparing between WG-seq and RAD-seq and between different pool sizes, we also evaluated in our study an alternative and more cost effective method of pooling. We show that extracting DNA from a pool of individuals gives similar accuracy as normalized pooling of individually extracted DNA. This is in spite of significant size variation in this species, with up to three-fold difference between the largest and smallest individuals. This may be because the experimental noise in pool-seq is on the same order of magnitude, or greater, than the size variation. The use of non-normalized pooling allows reducing DNA extraction costs as well as labor.

Previous studies investigated biases in pool-seq by computer simulations as well as empirical measurement of the error in allele frequency estimation from pool-seq relative to individual genotyping. Using simulation studies, Futschik and Schlötterer [19] concluded that lager pool sizes increase the accuracy of pool-seq, whereas here we report based on empirical measurements that pools of 10 individuals give similar results as pools of 30 individuals. This difference may stem from various types of experimental noise not modeled in the simulations (in DNA quantification, pipetting, PCR, and other stages of library construction, sequencing, and bioinformatic analysis). Van Tassell, et al. [16] and Gautier, et al. [14] empirically evaluated the accuracy of pool-seq combined with RAD-seq. Van Tassell, et al. [16] assessed the concordance of the genotypes of 66 individually sequenced samples with allele frequencies estimated by sequencing their pooled DNA. They reported a correlation coefficient *rho* = 0.67, which is similar to our *rho* = 0.68–0.75 for the RAD sequenced pools. Gautier, et al. [14] obtained a much better correlation with *rho* values between 0.93 and 0.99, depending on the depth of the loci in both pool and individual samples. The difference between the two studies may be due to the much greater sequencing depth of the pool, which was 191X in Gautier et al. but only 22X in Van Tassel et al. and 19X in our RAD-seq pools. Another important difference was that Gautier et al. created ten replicates of the pool, which reduces the effect of uneven individual representation in each pool if this noise is unbiased. Zhu, et al. [17] evaluated whole-genome pool-seq using pools of *Drosophila* isogenic lines with known genotypes. The largest difference in correlation was between the pool that sequenced at 10X depth (*rho* = 0.82–0.87) and other pools sequenced at 20X or more (*rho* > 0.9). Further differences between pools of more individuals (22 to 90) or greater depth (40X) were less pronounced (*rho* = 0.91–0.95). These previous empirical results are roughly in agreement with our results when comparing pool-seq with similar depth. With respect to the number of samples in the pool, previous studies reported somewhat increased accuracy with increased pool size. Conversely, our results show that 10 samples are sufficient to represent a colony, because this pool gave comparable accuracy to a pool of 30 samples. This result may be explained by the relatedness of individuals in the pools. In our study the pooled samples are either sisters or half-sisters, whereas previous studies used pools of unrelated samples.

### Conclusions

We conclude that for a given budget, pool-seq is preferable over individual sequencing for maximizing the QTL detection power in GWAS of colony level phenotypes. However, selection of library construction and sequencing approaches should consider the expected environmental noise, availability of samples, and manpower. We also conclude that when using pool-seq, sequencing whole genomes is preferable over sequencing reduced representation libraries and that, at least in social insects’ colonies, increasing the size of the sequenced pool is of little advantage. We also found that sequencing un-normalized pools may be of great advantage as it increases the number of colonies that can be analyzed, the power of GWAS, and reduces labor dramatically.

## Supporting information

S1 Table DNA concentration (ng/ul) in individually extracted samples. (DOCX)

S2 Table The effect of reduced sequencing depth on allele frequency accuracy in pool-seq (measured in terms of correlation between allele frequencies in individual genotypes and pool-seq). (DOCX)

S1 Fig Percent of phenotypic variation explained by the QTL for different levels of simulated environmental noise. (TIFF)
