# Supplementary material for: Comparative study of population genomic approaches for mapping colony-level traits
Source: PLoS Comput Biol. 2020 Mar 27;16(3):e1007653. doi: 10.1371/journal.pcbi.1007653 (PMC7141688; doi:10.1371/journal.pcbi.1007653)
Supplement: S1 Table — (DOCX) [file pcbi.1007653.s001.docx]

**Table S1: DNA concentration (ng/ul) in individually extracted samples**

| colony 48 | colony 49 | colony 50 |
| --- | --- | --- |
| 21.5 | 19.8 | 24.5 |
| 46.6 | 14.2 | 117.2 |
| 33.3 | 48.9 | 11.4 |
| 74.4 | 23.5 | 10.3 |
| 33.0 | 118.0 | 39.1 |
| 57.9 | 84.0 | 19.0 |
| 53.3 | 20.8 | 93.7 |
| 67.8 | 90.9 | 34.1 |
| 39.2 | 46.6 | 23.2 |
| 64.8 | 21.0 | 39.0 |
| 48.5 | 137.0 | 141 |
| 17.5 | 52.9 | 35.9 |
| 95.6 | 28.5 | 15.0 |
| 43.0 | 23.3 | 20.0 |
| 20.3 | 35.8 | 23.0 |
| 67.1 | 11.8 | 17.5 |
| 7.8 | 26.1 | 32.7 |
| 26.5 | 26.6 | 13.1 |
| 72.4 | 20.8 | 60.5 |
| 20.8 | 46.4 | 21.7 |
| 46.3 | 56.5 | 17.1 |
| 90.1 | 47.9 | 89.4 |
| 28.0 | 93.6 | 20.8 |
| 72.5 | 18.8 | 60.2 |
| 31.5 | 24.8 | 144.9 |
| 102.0 | 34.0 | 25.4 |
| 28.2 | 25.4 | 38.0 |
| 36.9 | 38.0 | 32.6 |
| 41.6 | 27.1 |  |
| 11.7 | 10.0 |  |
|  | 54.6 |  |
| Means | | |
| 46.67 | 42.43 | 43.58 |
| Standard deviations | | |
| 25.10 | 31.85 | 38.38 |
