# Supplementary material for: Comparative study of population genomic approaches for mapping colony-level traits
Source: PLoS Comput Biol. 2020 Mar 27;16(3):e1007653. doi: 10.1371/journal.pcbi.1007653 (PMC7141688; doi:10.1371/journal.pcbi.1007653)
Supplement: S2 Table — (DOCX) [file pcbi.1007653.s002.docx]

**Table S2: The effect of reduced sequencing depth on allele frequency accuracy in pool-seq (measured in terms of correlation between allele frequencies in individual genotypes and pool-seq)**

| Pool type | Depth reduction | Number of SNPs | Correlation |
| --- | --- | --- | --- |
| WG-seq normalized pool-10-1 | 2/3  1/2  1/3 | 181,189  22,922  12,095 | 0.914  0.820  0.759 |
| WG-seq normalized pool-10-2 | 2/3  1/2  1/3 | 181,242  22,919  12,089 | 0.908  0.817  0.756 |
| WG-seq normalized pool-30 | 2/3  1/2  1/3 | 180,370  22,820  12,084 | 0.914  0.824  0.766 |
| WG-seq  unnormalized pool-legs | 2/3  1/2  1/3 | 179,073  22,634  12,086 | 0.913  0.838  0.786 |
