# Supplementary figures and images for: Comparative study of population genomic approaches for mapping colony-level traits
Source: PLoS Comput Biol. 2020 Mar 27;16(3):e1007653. doi: 10.1371/journal.pcbi.1007653 (PMC7141688; doi:10.1371/journal.pcbi.1007653)

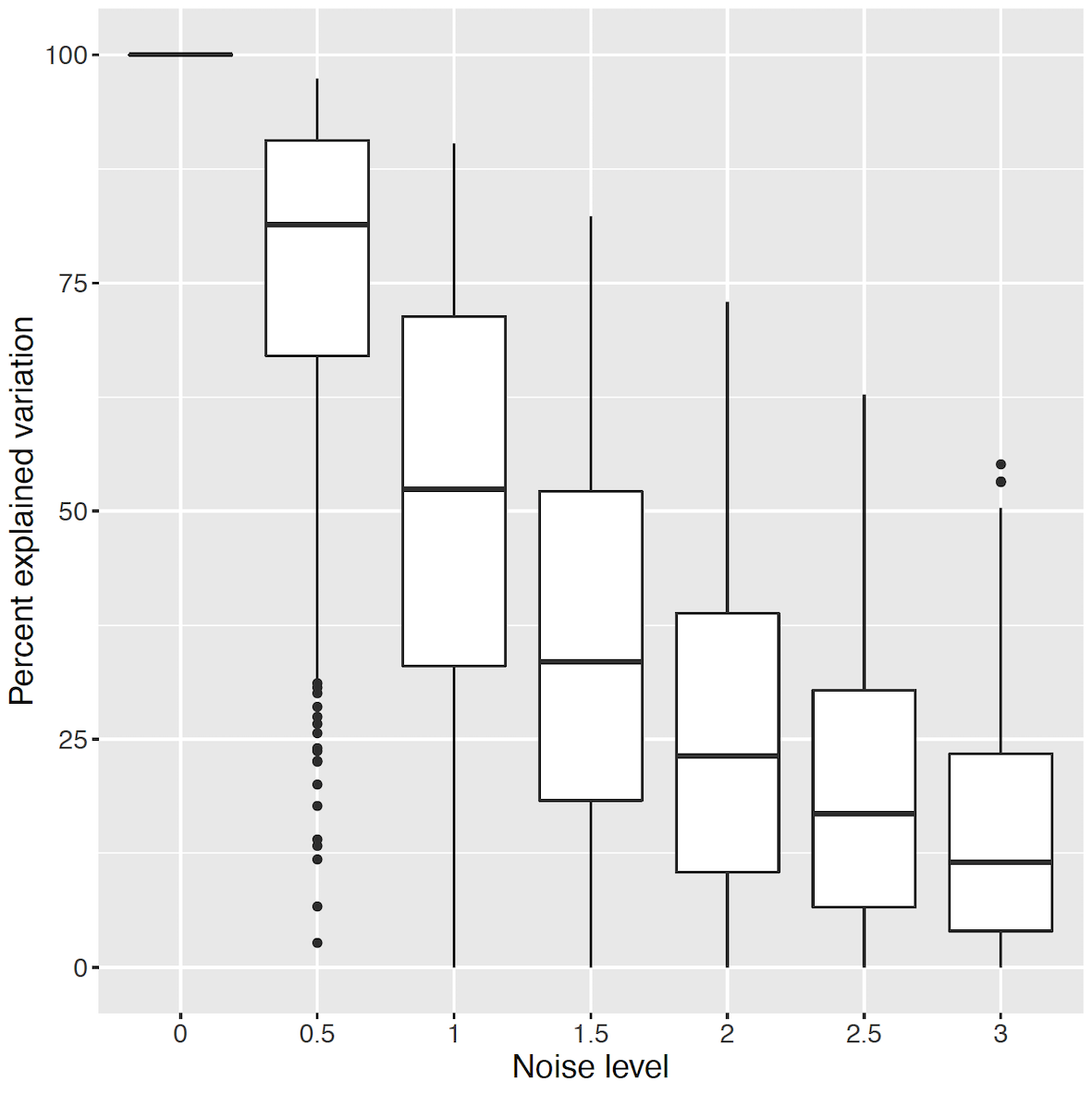

Supplement: S1 Fig — (TIFF) [file pcbi.1007653.s003.tiff]
